# Carotid Artery Temperature Reduction with Statin Therapy in Patients with Familial Hyperlipidemia Syndromes

**DOI:** 10.3390/jcm10215008

**Published:** 2021-10-27

**Authors:** Georgios Benetos, Spyros Galanakos, Iosif Koutagiar, Ioannis Skoumas, Georgios Oikonomou, Maria Drakopoulou, Maria Karmpalioti, Vasiliki Katsi, Costas Tsioufis, Konstantinos Toutouzas

**Affiliations:** 1 First Department of Cardiology, National & Kapodistrian University of Athens, 11527 Athens, Greece; sgalanakos@hotmail.com (S.G.); pepescutajar@hotmail.com (I.K.); skoumasj@yahoo.gr (I.S.); geooik88@gmail.com (G.O.); mdrakopoulou@hotmail.com (M.D.); karmpaliotimaria@gmail.com (M.K.); vkkatsi@yahoo.gr (V.K.); ktsioufis@gmail.com (C.T.); ktoutouz@gmail.com (K.T.); 2 First Department of Cardiology, Hygheia Hospital, 15123 Marousi, Greece

**Keywords:** microwave radiometry, carotid, vulnerable plaque, familial hypercholesterolemia

## Abstract

Background: Microwave radiometry (MWR) assesses non-invasive carotid artery temperatures reflecting inflammation. In the present study, we aimed to investigate the impact of hypolipidemic therapy either with simvastatin or with combination simvastatin plus ezetimibe on carotid artery temperatures of patients with familial hyperlipidemia syndromes (FHS). Methods: Consecutive patients with diagnosis of either familial heterozygous hypercholesterolemia (heFH) or familial combined hyperlipidemia (FCH) were included in the study. Patients were assigned to either simvastatin 40 mg or simvastatin 40 mg plus ezetimibe 10 mg, according to the discretion of the physician. FHS patients who refused statin therapy were used as a control group. Common carotid intima-media thickness (ccIMT) was measured and ΔΤ (maximum-minimum) temperature measurements were performed across each carotid during MWR evaluation. RESULTS: In total, 115 patients were included in the study. Of them, 40 patients received simvastatin (19 heFH and 21 FCH), 41 simvastatin + ezetimibe (31 heFH and 10 FCH), and 34 (21 heFH and 13 FCH) no statin. Carotid artery temperatures were significantly reduced at 6 months in FH patients who received hypolipidemic treatment (0.83 ± 0.34 versus 0.63 ± 0.24 °C, *p* = 0.004 for simvastatin, 1.00 ± 0.38 versus 0.69 ± 0.23 °C, *p* < 0.001 for simvastatin + ezetimibe), but no change was recorded in controls (0.72 ± 0.26 versus 0.70 ± 0.26 °C, *p* = 0.86). Conclusions: Hypolipidemic therapy reduced carotid temperatures in FHS patients.

## 1. Introduction

Statin therapy constitutes the main choice for the management of patients with familial hyperlipidemia syndromes. Nevertheless, statin therapy may be inadequate to achieve target LDL levels in cases of statin intolerance or very high pretherapy LDL values. The addition of ezetimibe has been introduced in clinical practice in such cases.

Robust data confirm the pleiotropic effect of statin therapy on atherosclerotic disease. It has been reported that statin therapy reduces carotid inflammation, as assessed by 18F-fluorodeoxyglucose positron emission tomography/computed tomography 18F-FDG uptake [1]. Indeed, atorvastatin therapy reduced atherosclerotic inflammation in a dose-dependent manner. According to previous studies, both heterozygous familial hypercholesterolemia (heFH) and familial combined hyperlipidemia (FCH) patients exhibit higher systemic as well as vascular inflammation, compared to controls [2], and maintained an inflammatory phenotype despite long-term therapy with statins [3].

Microwave radiometry (MWR) is a non-invasive and safe diagnostic tool, which allows in vivo evaluation of carotid inflammation, by measuring internal temperatures of tissues [4,5,6,7,8,9]. In previous studies, MWR measurements have shown good correlation with FDG-uptake values of carotid arteries in patients undergoing endarterectomy [10].

To our knowledge, current data regarding the pleiotropic effect of ezetimibe are scarce, [11] especially in familial hyperlipidemia syndromes. Moreover, there are no studies evaluating the mid-term effect of statin therapy ± ezetimibe on MWR-derived carotid temperatures.

In the present study, we aimed to evaluate whether (1) the initiation of statin therapy in patients with familial hyperlipidemia syndromes has an effect on carotid temperatures and (1) the addition of ezetimibe on top of simvastatin reduces incrementally carotid inflammation as estimated by measuring carotid temperatures.

## 2. Materials and Methods

### 2.1. Study Population

The patient population was recruited from the Lipid Outpatient Clinic of First Department of Cardiology of National & Kapodistrian University of Athens. Consecutive treatment-naïve patients with heFH or FCH, visiting the outpatient clinic from April 2015 to January 2019, were prospectively recruited and were assigned to therapy either with simvastatin 40 mg or with combination simvastatin 40 mg plus ezetimibe 10 mg. The assignment was left to the discretion of the attending physician. Patients with familial hyperlipidemia syndromes who denied any hypolipidemic treatment (including PCSK inhibitors) were also included in a separate registry. Exclusion criteria for the dyslipidemic population were hypolipidemic treatment in the past 6 months, known cardiovascular disease, active infections, active cancer, inflammatory or active autoimmune disease under corticosteroid and/or NSAID therapy, end-stage renal disease, chronic liver disease, and a life expectancy <2 years.

All patients provided written, informed consent for the participation in the study. The study was approved by the institutional Ethics committee and conformed to the Declaration of Helsinki.

### 2.2. Data Collection at Baseline

Weight and height were measured in all participants for calculation of body mass index (BMI). All cardiovascular risk factors, including hypertension, smoking, diabetes mellitus, and family history of premature coronary artery disease (CAD), were recorded. Total cholesterol, HDL-cholesterol, triglycerides, and high sensitivity c-reactive protein (hs-CRP) were measured after an overnight fasting, as previously described [1]. Total cholesterol, HDL-C, and triglycerides were measured using the colorimetric enzymic method in a Technicon automatic analyzer RA-1000 (Dade-Behring Marburg GmbH, Marburg, Germany). LDL cholesterol was calculated by the Friedewald formula unless patients had triglycerides level >400 mg/dL.

### 2.3. Diagnosis of heFH and FCH

Clinical diagnosis of heFH was based on the Dutch Lipid clinical criteria. In specific, individuals with a Dutch Lipid Criteria score > 5 were included in the study [2].

The diagnosis of FCH was established when the patient and at least one family member had plasma triglyceride levels more than 133 mg/dL and apoB more than 120 mg/dL, according to previously published criteria [3]. Moreover, the presence of a family history of premature cardiovascular disease was confirmatory of the diagnosis.

### 2.4. Carotid Ultrasound Measurements

A high-resolution B-mode ultrasound unit (iE33 xMATRIX, Philips Healthcare, Bothell, WA, USA) with a 7.5-MHz transducer was used to examine both carotid arteries in transverse and longitudinal sections. Ultrasound imaging methods have been previously described [4,5,6,7]. Intima media thickness in a common carotid artery (cc-IMT) and carotid plaque thickness measurements were performed according to the Mannheim consensus [8]. In specific, cc-IMT was measured over the last 10 mm of the distal wall of both common carotids at a region without plaque. The highest value of ccIMT for both carotid arteries was assigned as ccIMTmax. Similarly, plaque thickness was measured in each detectable plaque. An experienced sonographer (G.B.) performed all ultrasound measurements.

### 2.5. Microwave Radiometry Measurements

A microwave computer-based system (RTM 01 RES, Bolton, UK) that detects temperature from internal tissues at microwave frequencies was used to measure the carotid plaque temperature. The application principles of the device have been previously described [4,5,6,7,9,10,11,12,13]. In brief, MWR measurements were obtained over the abovementioned segments, at room temperature between 20 and 24 °C, at least 10 min after the ultrasound examination, in order to avoid any influence on temperature from palpation or the ultrasound study. After setting the transducer vertically in touch with the skin, carotid temperature measurements were performed three times on each segment to assess the intraobserver reproducibility of the method (overall, nine measurements). The temperature of each segment used for further analysis was the mean of the three temperatures. This procedure was repeated over the three previously defined from the ultrasound segments, starting from the distal to the proximal. Temperature difference (DT) was then defined as maximal temperature detected along the carotid artery minus minimum. The method was validated, as previously described [7]. All MWR measurements were obtained by an experienced physician (I.K.).

### 2.6. Follow-up Measurements

All familial hyperlipidemia patients were followed up clinically. Carotid ultrasound, MWR, lipid profile, and CRP measurements were repeated at 6 months.

### 2.7. Statistical Analysis

Statistical analysis was performed with commercially available software (SPSS, version 22, SPSS Inc., Chicago, IL, USA). Continuous variables are presented as rates or mean values ± SD, while categorical variables, as absolute and relative frequencies. Probability values are two-sided from the Student’s *t*-test or the Mann–Whitney U test for continuous variables, according to the normal or skewed distribution of the variables. Assessment for normality of data distribution was evaluated by the Kolmogorov–Smirnov test. For multiple groups’ comparisons, ANOVA or Kruskal–Wallis test was used. A *p*-value <0.05 was considered statistically significant. Moreover, Bonferroni correction was applied for multiple between-group comparisons. Changes in ΔT values of the same patients between baseline and follow-up were examined by paired *t* test. Pearson’s correlation coefficient was used to analyze the correlation of changes in DT and cholesterol values. A two-tailed value of *p* <0.05 was considered statistically significant.

## 3. Results

### 3.1. Study Population

**Figure 1:**
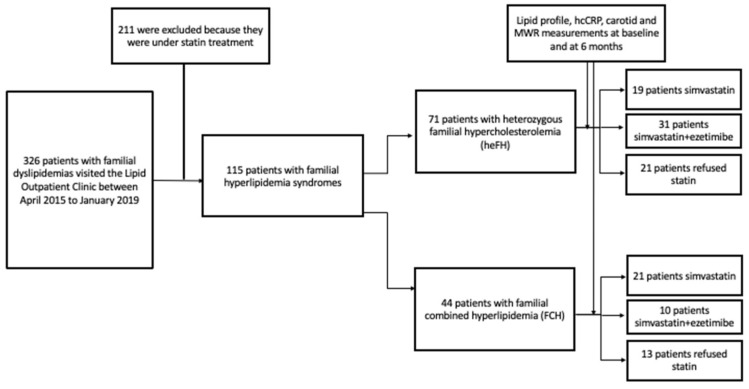
Study flow chart.

In total, 115 patients with familial hyperlipidemia syndromes were included in the study. Of them, 71 patients had heFH and 44 patients had FCH (Figure 1). Among hyperlipidemic patients, 40 (19 heFH and 21 FCH) were assigned to simvastatin therapy, 41 (31 heFH and 10 FCH) to simvastatin + ezetimibe therapy, and 34 refused statin therapy. The baseline characteristics are summarized in Table 1.

In total, nine patients had carotid plaques. Of them, six patients had heFH and three had FCH.

### 3.2. Changes from Baseline of ccIMT, DT, and Laboratory Parameters, Stratified According to Assigned Therapy

There was a statistically significant reduction at 6 months of cc-IMT measurements in patients assigned both to simvastatin (1.0 ± 0.3 versus 0.9 ± 0.1 mm, *p* = 0.04) and simvastatin + ezetimibe therapy (1.01 ± 0.3 versus 0.9 ± 0.2 mm, *p* < 0.001). In contrast, no significant difference was noted in patients without statin therapy (1.0 ± 0.2 versus 0.9 ± 0.2 mm, *p* = 0.06).

Regarding carotid temperatures, these were significantly reduced after 6 months of simvastatin (0.83 ± 0.34 versus 0.63 ± 0.24 °C, *p* = 0.004) or simvastatin + ezetimibe therapy (1.00 ± 0.38 versus 0.69 ± 0.23 °C, *p* < 0.001). No change was noted in patients without statin (0.72 ± 0.26 versus 0.70 ± 0.26 °C, *p* = 0.86).

Lastly, there were no significant changes between baseline and follow-up hsCRP measurements for all three groups (no statin: 1.43 ± 1.4 versus 1.75 ± 2.31 mg/dL, *p* = 0.54; simvastatin: 2.66 ± 2.65 versus 2.98 ± 4.62 mg/dL, *p* = 0.73; and simvastatin + ezetimibe: 2.08 ± 2.78 versus 1.40 ± 1.30, *p* = 0.12).

**Figure 2:**
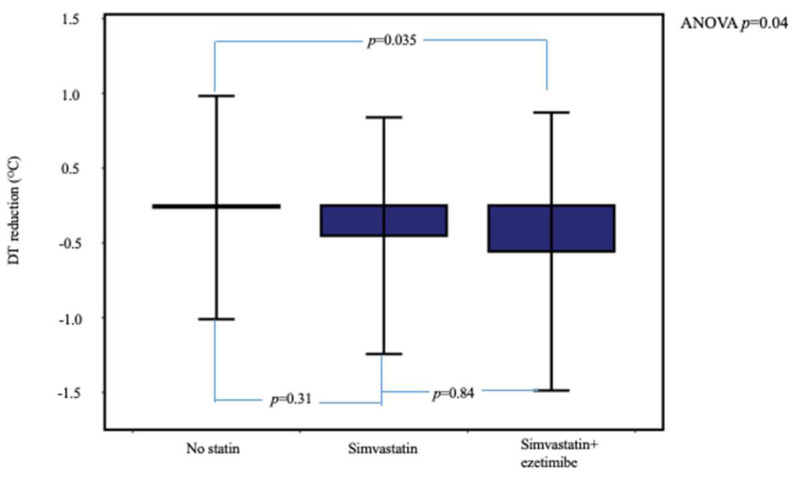
Temperature reduction at 6 months, stratified according to the assigned therapy. Patients under simvastatin + ezetimibe therapy showed higher DT reduction. Error bars show ± 2SD.

In Table 2 is compared the range of 6-month changes in ccIMT, DT, and laboratory parameters between the three patient groups. Temperature reduction was significantly higher in dyslipidemic patients under statin therapy compared to the no-statin group (Table 2 and Figure 2). In post hoc between-group analysis, the difference remained significant between the simvastatin + ezetimibe and no-statin groups (*p* = 0.035) but not between the simvastatin and simvastatin + ezetimibe groups (*p* = 0.84).

### 3.3. Changes from Baseline of ccIMT, DT, and Laboratory Parameters in FCH Patients Stratified According to Assigned Therapy

The cc-IMT measurements were significantly reduced at 6 months in the no-statin group of FCH patients (1.06 ± 0.23 versus 0.84 ± 0.21 mm, *p* = 0.006) but not in the simvastatin or simvastatin + ezetimibe groups (0.95 ± 0.15 versus 0.93 ± 0.14 mm, *p* = 0.58 and 1.06 ± 0.37 versus 0.96 ± 0.22 mm, *p* = 0.30, respectively).

Carotid temperatures were significantly lower at follow-up in simvastatin-treated FCH patients (0.81 ± 0.30 versus 0.63 ± 0.26 °C, *p* = 0.03). In simvastatin + ezetimibe-treated FCH patients, carotid temperatures were also lower at follow-up, although not reaching statistical significance (1.01 ± 0.39 versus 0.74 ± 0.21 °C, *p* = 0.12). In addition, no significant change was recorded in carotid temperatures in FCH patients who did not receive any statin (0.77 ± 0.31 versus 0.62 ± 0.19 °C, *p* = 0.28).

Lastly, there were no significant changes between baseline and follow-up hsCRP measurements for all three treatment groups of FCH patients (no-statin: 2.17 ± 1.49 versus 2.81 ± 2.94 mg/dL, *p* = 0.59; simvastatin: 3.06 ± 2.73 versus 3.54 ± 5.43 mg/dL, *p* = 0.73; and simvastatin+ezetimibe: 3.65 ± 3.97 versus 2.30 ± 1.64 mg/dL, *p* = 0.31).

In Table 3 is compared the range of 6-month changes in ccIMT, DT, and laboratory parameters in the FCH subgroup patients according to the assigned therapy. The difference in carotid temperature reduction was not statistically significant between the patient groups.

### 3.4. Changes from Baseline of ccIMT, DT, and Laboratory Parameters in heFH Patients Stratified According to Assigned Therapy

Carotid IMT was significantly lower at follow-up in both simvastatin- (1.05 ± 0.45 versus 0.86 ± 0.15 mm, *p* = 0.04) and simvastatin + ezetimibe (1.07 ± 0.32 versus 0.89 ± 0.20 mm, *p* < 0.001)-treated heFH patients, but not in heFH patients who did not receive statin (0.94 ± 0.24 versus 0.92 ± 0.24 mm, *p* = 0.81).

A significant carotid temperature reduction was recorded in simvastatin + ezetimibe-treated heFH patients (1.00 ± 0.38 versus 0.68 ± 0.24 °C, *p* = 0.001). In contrast, the reduction in the simvastatin-treated group did not reach statistical significance (0.85 ± 0.39 versus 0.63 ± 0.22 °C, *p* = 0.06). There was no significant difference in carotid temperatures between baseline and follow-up in the no-statin heFH patients (0.67 ± 0.21 versus 0.78 ± 0.30 °C, *p* = 0.26).

Lastly, there were no significant changes between baseline and follow-up hsCRP measurements for all three treatment groups of heFH patients (no-statin: 0.78 ± 0.99 versus 0.83 ± 1.07 mg/dL, *p* = 0.65; simvastatin: 1.81 ± 2.41 versus 1.80 ± 1.85 mg/dL, *p* = 0.99; and simvastatin+ezetimibe: 1.39 ± 1.79 versus 1.00 ± 0.91 mg/dL, *p* = 0.19).

In Table 4 is compared the range of 6-month changes in ccIMT, DT, and laboratory parameters in the heFH subgroup patients according to the assigned therapy. In this patient subgroup, carotid temperature reduction was significantly higher in the simvastatin+ezetimibe group compared to the simvastatin or no-statin groups.

### 3.5. Changes from Baseline of DT and Laboratory Parameters, Stratified According to heFH or FCH

Regarding carotid artery temperatures, statin therapy led to a reduction in both dyslipidemic groups (heFH: 0.95 ± 0.39 versus 0.66 ± 0.23 °C, *p* < 0.001 and FCH: 0.87 ± 0.33 versus 0.66 ± 0.25 °C, *p* = 0.005).

Lastly, there were no significant changes in hsCRP measurements between baseline and follow-up for both groups (heFH: 1.53 ± 1.98 versus 1.27 ± 1.32 mg/dL, *p* = 0.40 and FCH: 3.23 ± 3.08 versus 3.17 ± 4.63 mg/dL, *p* = 0.95).

In Table 5 are compared the baseline characteristics of heFH and FCH patients assigned to statin therapy together with the range of 6-month changes. Briefly, ccIMT reduction was higher in heFH, compared to the FCH group. Carotid temperature reduction, however, was similar between the abovementioned groups.

### 3.6. Correlations between Cholesterol and Temperature Changes

There was a statistically significant correlation between DT reduction and cholesterol reduction in dyslipidemic patients under statin therapy (R = 0.32, *p* = 0.003). In contrast, no correlation was noted between LDL and DT reduction (R = 0.13, *p* = 0.29).

## 4. Discussion

In the present study, we showed that (a) hypolipidemic therapy significantly reduced carotid temperatures in both heFH and FCH patients but there were no indices of systemic inflammation and (b) the addition of ezetimibe to simvastatin therapy had no incremental impact on carotid temperature.

To our knowledge, this is the first study to evaluate the impact of statin therapy +/− ezetimibe on carotid temperatures of patients with familial hyperlipidemia syndromes.

Our findings suggest that statin therapy, either alone or in combination with ezetimibe, significantly reduced carotid temperatures in those patients. This is in line with recent studies, where both statin monotherapy and the combination of ezetimibe with either simvastatin or rosuvastatin reduced FDG carotid wall uptake in patients with recent acute coronary syndrome [14,15].

Although robust data exist about the impact of statin therapy on carotid wall inflammation [16,17,18], few data exist regarding the impact of ezetimibe on top of statin therapy on arterial wall inflammatory activation. In specific, the combination of ezetimibe with statin, compared to statin monotherapy, exerts a similar anti-inflammatory effect on carotid atherosclerotic plaque [14,15]. It is noteworthy that in a recent study the combination of ezetimibe with low-dose rosuvastatin showed inferior carotid wall anti-inflammatory effect compared to high-dose rosuvastatin [19]. Nevertheless, all the above studies did not investigate the incremental anti-inflammatory impact of ezetimibe, but rather compared the combination of ezetimibe/statin to a statin dose with a similar LDL-lowering effect.

In accordance with the results of the PRECISE-IVUS study, in the present study hypolipidemic treatment did not reduce significantly the value of circulatory inflammatory biomarkers [20]. It has been shown that statin therapy lowers CRP and/or circulating pro-inflammatory cytokines’ levels in patients with hypercholesterolemia [21,22] and familial combined hyperlipidemia [23]. In addition, in previous studies with heFH individuals, the mid- or long-term combined therapy of simvastatin/ezetimibe reduced significantly the CRP levels [24,25]. Although these data suggest that ezetimibe could exert anti-inflammatory effects, it remains unclear the additive anti-inflammatory effect of ezetimibe and whether this effect is independent of a LDL cholesterol-lowering result [26].

In the present study, statin therapy, either alone or in combination with ezetimibe, significantly reduced carotid artery temperatures in patients with familial hyperlipidemia syndromes. The same was also true in both the heFH and FCH subgroups More importantly, the evaluation of carotid artery temperatures was achieved by the means of microwave radiometry. Thus, microwave radiometry as a noninvasive method without radiation exposure could represent a promising tool for the monitoring of patients with familial hypercholesterolemia.

Clinical implications: In previous studies by our group, it was shown that Carotid temperatures as assessed by microwave radiometry have prognostic value [5,6]. Indeed, higher temperatures in patients with coronary artery disease have been associated with increased rates of cardiovascular events. Thus, carotid temperature monitoring in the high-risk population of familial hyperlipidemia syndromes is also of clinical importance.

Future directions: As recent data failed to confirm an undoubtable anti-inflammatory effect of ezetimibe, the anti-inflammatory effects of a new, promising hypolipidemic therapy, the PCSK9 inhibition may be an area of future interest. Considering that, according to recent studies, PCSK9 inhibitors did not reduce CRP levels [27], PCSK9 inhibition may modify other aspects of the inflammatory response than statins. Moreover, the anti-inflammatory potential of the combination of these drugs should be further investigated.

### Limitations

This was a single-center study and all patients were recruited from one Lipid Outpatient Clinic. Moreover, the assignment of simvastatin or simvastatin + ezetimibe therapy to patients with familial hyperlipidemia syndromes was left to the discretion of the physician, according to baseline LDL levels. MWR and IMT measurements were performed by a single operator. Baseline between-group temperature differences were present. However, paired temperature comparisons revealed temperature reduction only in the statin treatment groups. Lastly, the paradox of ccIMT reduction only in the subgroup of FCH patients without statin and not in the statin group could be attributed to the low number of patients.

## 5. Conclusions

Hypolipidemic therapy significantly reduced carotid artery temperatures in patients with familial hyperlipidemia syndromes. The addition of ezetimibe on top of simvastatin resulted in no significant incremental benefit.

## Figures and Tables

**Table 1:** Baseline characteristics of the study population.

	No Statin (*n* = 34)	Simvastatin (*n* = 40)	Simvastatin + Ezetimibe (*n* = 41)	*p*
Age	40.32 ± 12.35	43.95 ± 12.81	41.98 ± 14.07	0.5
Male—*n* (%)	22 (64.7)	26 (65)	23 (56.1)	0.65
BMI (kg/m^2^)	26.22 ± 4.39	27.25 ± 4.84	27.10 ± 4.91	0.61
Smoking—*n* (%)	15 (44.1)	19 (47.5)	21 (51.2)	0.83
Hypertension—*n* (%)	5 (14.7)	12 (30)	6 (14.6)	0.15
Family History of CAD—*n* (%)	9 (26.5)	19(47.5)	16 (39)	0.18
Diabetes mellitus—*n* (%)	0 (0)	5 (12.5)	2 (4.9)	0.08
**Laboratory parameters**				
Total Cholesterol (mg/dL)	271.58 ± 39.44 * ^#^	289.68 ± 56.13	333.49 ± 100.29 * ^#^	0.001
LDL Cholesterol (mg/dL)	199.32 ± 40.34 ^$^	203.19 ± 44.35 ^	247.07 ± 96.71 ^$^ ^	0.005
HDL Cholesterol (mg/dL)	45.45 ± 11.02	47.80 ± 15.53	51.85 ± 15.57	0.16
TG (mg/dL)	186.39 ± 137.86	247.15 ± 224.78	165.49 ± 108.01	0.08
hsCRP (mg/dL)	1.70 ± 1.69	2.61 ± 2.54	1.96 ± 2.67	0.34
**Carotid IMT**				
Left Common carotid IMT (mm)	0.90 ± 0.20	0.93 ± 0.32	0.94 ± 0.23	0.8
Right Common carotid IMT (mm)	0.89 ± 0.23	0.89 ± 0.20	0.97 ± 0.03	0.36
Maximal Common carotid IMT (mm)	0.95 ± 0.23	0.99 ± 0.32	1.05 ± 0.33	0.36
**Carotid temperatures**				
Left DT °C	0.62 ± 0.25 ^&^	0.69 ± 0.31	0.86 ± 0.38 ^&^	0.005
Right DT °C	0.68 ± 0.34	0.74 ± 0.32	0.84 ± 0.37	0.13
Maximal DT °C	0.75 ± 0.33 ″	0.84 ± 0.33	0.99 ± 0.37 ″	0.01
**Baseline medication**				
Aspirin—*n* (%)	0 (0)	1 (9.5)	3 (7.3)	0.21
ACE inhibitors—*n* (%)	2 (5.9)	1 (2.5)	2 (4.9)	0.76
ATII receptor antagonists—*n* (%)	0 (0)	5 (12.5)	1 (2.4)	0.03
b-blockers—*n* (%)	1 (2.9)	2 (5)	3 (7.3)	0.7
Calcium antagonists—*n* (%)	0 (0)	3 (7.5)	2 (4.9)	0.28

BMI: Body Mass Index; CAD: coronary artery disease; hsCRP: high sensitivity CRP; ACE inhibitors: angiotensin converting enzyme inhibitors; ATII receptor antagonists: angiotensin II receptor antagonists. * Bonferroni *p* = 0.01, ^#^ *p* = 0.02, ^$^ *p* = 0.01, ^ *p* = 0.02, ^&^ *p* = 0.005, ″ *p* = 0.009.

**Table 2:** Comparison of carotid IMT, carotid temperature, and laboratory parameter changes at 6 months of follow-up between study groups.

Difference from Baseline at 6 Months	No Statin (*n* = 34)	Simvastatin (*n* = 40)	Simvastatin + Ezetimibe (*n* = 41)	*p*
Left Common carotid IMT (mm)	−0.08 ± 0.2	−0.1 ± 0.3	−0.09 ± 0.2	0.88
Right Common carotid IMT (mm)	−0.01 ± 0.2	−0.02 ± 0.2	−0.14 ± 0.3	0.08
Maximal Common carotid IMT (mm)	−0.1 ± 0.2	−0.09 ± 0.3	−0.16 ± 0.3	0.52
Left DT °C	0.01 ± 0.3	−0.14 ± 0.35	−0.29 ± 0.45	0.02
Right DT °C	−0.005 ± 0.4	−0.16 ± 0.36	−0.23 ± 0.47	0.14
Maximal DT °C	−0.01 ± 0.37	−0.20 ± 0.40	−0.31 ± 0.46	0.04
Total Cholesterol (mg/dL)	−17.85 ± 26.21	−113.26 ± 54.80	−153.22 ± 95.10	<0.001
LDL Cholesterol (mg/dL)	−22.27 ± 28.63	−69.39 ± 68.10	−128.75 ± 80.92	<0.001
HDL Cholesterol (mg/dL)	1.38 ± 8.66	−1.72 ± 8.66	−2.97 ± 8.33	0.31
TG (mg/dL)	−22.08 ± 64.03	−93.51 ± 158.51	−62.83 ± 75.25	0.07
hsCRP (mg/dL)	0.18 ± 2.03	0.32 ± 4.96	−0.67 ± 2.17	0.56

**Table 3:** Comparison of carotid IMT, carotid temperature, and laboratory parameter changes at 6 months of follow-up in FCH subgroup.

FCH (*n* = 44)	No Statin (*n* = 13)	Simvastatin (*n* = 21)	Simvastatin + Ezetimibe (*n* = 10)	*p*
**Difference from baseline at 6 months**				
Left Common carotid IMT (mm)	−0.13 ± 0.08	−0.05 ± 0.15	−0.07 ± 0.11	0.29
Right Common carotid IMT (mm)	−0.23 ± 0.19	0.02 ± 0.2	−0.08 ± 0.27	0.009
Maximal Common carotid IMT (mm)	−0.22 ± 0.18	−0.02 ± 0.13	−0.10 ± 0.27	0.03
Left DT °C	−0.06 ± 0.28	−0.12 ± 0.34	−0.41 ± 0.47	0.08
Right DT °C	−0.18 ± 0.47	−0.20 ± 0.28	0.03 ± 0.36	0.26
Maximal DT °C	−0.15 ± 0.41	−0.19 ± 0.34	−0.27 ± 0.46	0.78
Total Cholesterol (mg/dL)	−11.83 ± 18.15	−121 ± 64.66	−109.56 ± 64.65	0.002
LDL Cholesterol (mg/dL)	−19 ± 11.77	−50.14 ± 87.47	−83.25 ± 35.91	0.25
HDL Cholesterol (mg/dL)	2.17 ± 7.73	2.06 ± 6.76	−0.44 ± 9.02	0.70
TG (mg/dL)	−42.83 ± 87.27	−150.82 ± 196.41	−112.44 ± 109.77	0.38
hsCRP (mg/dL)	0.64 ± 3.02	−0.48 ± 5.88	−1.35 ± 3.52	0.65

**Table 4:** Comparison of carotid IMT, carotid temperature, and laboratory parameter changes at 6 months of follow-up in heFH subgroup.

heFH (*n* = 71)	No Statin (*n* = 21)	Simvastatin (*n* = 19)	Simvastatin + Ezetimibe (*n* = 31)	*p*
**Difference from Baseline at 6 Months**				
Left Common carotid IMT (mm)	−0.04 ± 0.26	−0.18 ± 0.39	−0.10 ± 0.19	0.38
Right Common carotid IMT (mm)	0.02 ± 0.25	−0.08 ± 0.20	−0.17 ± 0.26	0.1
Maximal Common carotid IMT (mm)	−0.02 ± 0.27	−0.19 ± 0.35	−0.18 ± 0.25	0.24
Left DT °C	0.07 ± 0.32	−0.16 ± 0.38	−0.26 ± 0.45	0.08
Right DT °C	0.15 ± 0.27	−0.12 ± 0.44	−0.32 ± 0.48	0.01
Maximal DT °C	0.11 ± 0.30	−0.22 ± 0.46	−0.32 ± 0.47	0.03
Total Cholesterol (mg/dL)	−23 ± 32.13	−104.50 ± 41.39	−167.78 ± 100	<0.001
LDL Cholesterol (mg/dL)	−25 ± 38.85	−88.64 ± 34.37	−143.31 ± 86.27	0.001
HDL Cholesterol (mg/dL)	0.7 ± 9.95	−6.6 ± 9.97	−3.81 ± 8.09	0.22
TG (mg/dL)	−4.29 ± 32.46	−23.93 ± 31.55	−46.30 ± 52.72	0.07
hsCRP (mg/dL)	−0.18 ± 0.73	−0.005 ± 2.23	−0.37 ± 1.23	0.82

**Table 5:** Comparison of baseline and follow-up characteristics between heFH and FCH patients under statin therapy.

Baseline Characteristics	heFH (*n* = 71)	FCH (*n* = 44)	*p*
Simvastatin	19 (26.8)	21 (47.7)	0.009
Simvastatin + ezetimibe	31 (43.7)	10 (22.7)	
No statin	21 (29.6)	13 (29.5)	
Left Common carotid IMT (mm)	0.96 ± 0.32	0.90 ± 0.18	0.38
Right Common carotid IMT (mm)	0.95 ± 0.31	0.90 ± 0.25	0.47
Maximal Common carotid IMT (mm)	1.05 ± 0.36	0.97 ± 0.24	0.24
Left DT °C	0.79 ± 0.35	0.76 ± 0.36	0.79
Right DT °C	0.83 ± 0.39	0.74 ± 0.25	0.26
Maximal DT °C	0.83 ± 0.39	0.74 ± 0.25	0.26
Total Cholesterol (mg/dL)	334.92 ± 89.50	274.65 ± 58.32	0.001
LDL cholesterol (mg/dL)	251.90 ± 85.46	179.63 ± 34.21	<0.001
HDL cholesterol (mg/dL)	57.24 ± 14.51	37.94 ± 8.14	<0.001
TG (mg/dL)	119.38 ± 55.39	345.23 ± 219.53	<0.001
hsCRP (mg/dL)	1.44 ± 1.88	3.2 ± 2.97	0.08
**Difference from baseline at 6 months**			
Left Common carotid IMT (mm)	−0.13 ± 0.27	−0.06 ± 0.14	0.19
Right Common carotid IMT (mm)	−0.14 ± 0.24	−0.01 ± 0.20	0.02
Maximal Common carotid IMT (mm)	−0.18 ± 0.29	−0.04 ± 0.19	0.02
Left DT °C	−0.22 ± 0.42	−0.21 ± 0.40	0.89
Right DT °C	−0.25 ± 0.47	−0.12 ± 0.32	0.23
Maximal DT °C	−0.28 ± 0.47	−0.21 ± 0.38	0.47
Total Cholesterol (mg/dL)	−144.23 ± 88.12	−117.22 ± 63.64	0.17
LDL cholesterol (mg/dL)	−123.69 ± 76.23	−62.18 ± 73.71	0.003
HDL cholesterol (mg/dL)	−4.76 ± 8.75	1.22 ± 7.51	0.004
TG (mg/dL)	−38.66 ± 47.38	−137.54 ± 169.97	0.001
hsCRP (mg/dL)	−0.25 ± 1.6	− 0.06 ± 5.29	0.86

## Data Availability

Not applicable.
